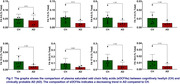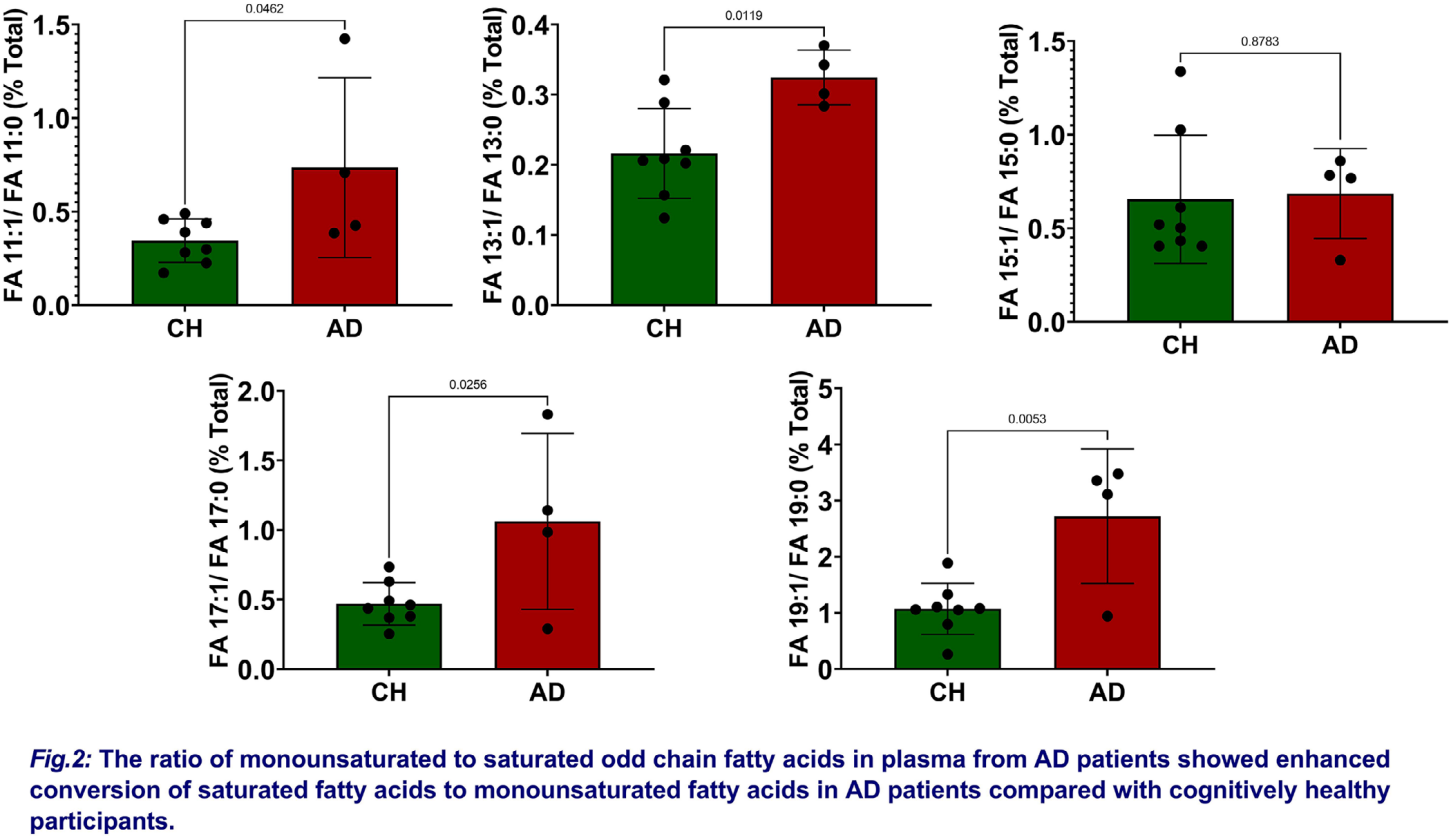# A lower plasma odd chain fatty acids composition accompanies a significant increase in the desaturase index in Alzheimer’s disease

**DOI:** 10.1002/alz.092138

**Published:** 2025-01-09

**Authors:** Joby Jose, Alfred N. Fonteh

**Affiliations:** ^1^ Huntington Medical Research Institutes, Pasadena, CA USA

## Abstract

**Background:**

Odd‐chain fatty acids (OCFA) are gut microbiota‐derived metabolites that are important in energy generation, neuronal signaling, and memory. Since the composition of the gut microbiota affects cognitive function, we hypothesize that plasma saturated OCFA composition may be altered in AD compared to cognitively healthy older adults.

**Method:**

Older adults (>65 years) were recruited, and demographic and neurological data obtained in an ongoing brain‐aging project. Participants were classified as cognitively healthy (CH) or clinically probable AD (AD) based on a comprehensive neuropsychological assessment. Overnight fasting plasma was collected, and unesterified fatty acids were extracted using acidified ethyl acetate. Plasma saturated OCFA (sOCFA: FA 5:0, FA 7:0, FA 9:0, FA 11:0, FA 13:0, FA 15:0, FA 17:0 & FA 19:0) and monounsaturated OCFA (mOCFA: FA 11:1, FA 13:1, FA 15:1, FA 17:1 & FA 19:1) compositions were measured using liquid chromatography tandem mass spectrometry after derivatization with dimethylaminophenacyl bromide (DmPABr). OCFA compositions were determined as a percentage (± SD, 95% CI) of 42 fatty acids detected in plasma, and the CH to AD differences were determined using Mann‐Whitney tests.

**Result:**

Eight sOCFAs (FA 5:0‐FA 19:0) and five mOCFAs (FA 11:1‐FA 19:1) were detected and quantified in plasma. The findings indicated a general trend of decreased sOCFA in AD plasma as compared to CH plasma (Figure 1). The total sOCFA composition in CH (0.24 ± 0.18, 0.09–0.39, n = 8) was higher than in AD (0.1 ± 0.07, ‐0.09–0.25, n= 4, p= 0.0545). The desaturase index (ratio of mOCFA to sOCFA) for FA:11, FA:13, FA:17, and FA:19 was significantly higher in AD than in CH (Figure 2). The total CH (0.52 ± 0.14, 0.41‐0.64) desaturase index was significantly lower (p< 0.05) than in AD (0.86 ± 0.32, 0.34‐1.38).

**Conclusion:**

These data suggest that AD participants have a lower plasma sOCFA composition, which may be due to enhanced conversion of sOCFA to mOCFA, as evidenced by a higher desaturase index in AD compared to CH. Therefore, measurement of plasma sOCFA can be used to monitor AD‐associated lipid metabolism and pathways linked to microbiome status.